# Isolation and complete genomic characterization of H1N1 subtype swine influenza viruses in southern China through the 2009 pandemic

**DOI:** 10.1186/1743-422X-8-129

**Published:** 2011-03-20

**Authors:** Yizhi Liu, Jun Ji, Qingmei Xie, Jing Wang, Huiqin Shang, Cuiying Chen, Feng Chen, Chunyi Xue, Yongchang Cao, Jingyun Ma, Yingzuo Bi

**Affiliations:** 1 College of Animal Science, South China Agricultural University, Guangzhou 510642, PR China; 2 Guangdong Wen's Foodstuffs Group Co. Ltd., Yunfu 527439, PR China; 3 State Key Laboratory of Biocontrol, College of Life Sciences, Sun Yat-Sen University, Guangzhou 510006, PR China

## Abstract

**Background:**

The swine influenza (SI) is an infectious disease of swine and human. The novel swine-origin influenza A (H1N1) that emerged from April 2009 in Mexico spread rapidly and caused a human pandemic globally. To determine whether the tremendous virus had existed in or transmitted to pigs in southern China, eight H1N1 influenza strains were identified from pigs of Guangdong province during 2008-2009.

**Results:**

Based on the homology and phylogenetic analyses of the nucleotide sequences of each gene segments, the isolates were confirmed to belong to the classical SI group, with HA, NP and NS most similar to 2009 human-like H1N1 influenza virus lineages. All of the eight strains were low pathogenic influenza viruses, had the same host range, and not sensitive to class of antiviral drugs.

**Conclusions:**

This study provides the evidence that there is no 2009 H1N1-like virus emerged in southern China, but the importance of swine influenza virus surveillance in China should be given a high priority.

## Background

Swine influenza (SI) is an acute respiratory disease caused by influenza A virus, a member of the *Orthomyxoviridae *family. Influenza A viruses have been isolated from a number of animals including humans, birds, dogs, seals, horses and swine [1]. The causative agent is RNA virus with a segmented genome comprised of eight negative-sense, single-stranded RNA segments encoding eleven proteins [2].

Hemagglutinin (HA) and neuraminidase (NA) are two major surface glycoproteins of influenza viruses and important for host range, antigenicity, pathogenesis and diagnostic detection. Influenza A viruses are classified into a number of subtypes based on antigenic differences in haemagglutinin (HA; 16 subtypes) and neuraminidase (NA; 9 subtypes) [3,1]. Although other subtypes have been sporadically identified, there are three major subtypes of SIV (H1N1, H1N2, and H3N2) circulating in the swine population throughout the world currently [4,5].

Pigs have the susceptibility of infecting avian and human influenza, for the cells respiratory tract express both sialic acid-a2,3-galactose (SAa2,3Gal) receptors and sialic acid-a2,6-galactose (SAa2,6Gal) receptors, preferred by avian influenza viruses and human influenza viruses, respectively [6,7]. Therefore, pigs play an important role in supporting co-infection, replication, and reassortment among human, avian, and swine influenza viruses, acting as an intermediate host or mixing vessels thereby creating novel reassortant viruses with pandemic potential [4,6,8].

Since Influenza was first described as a disease of swine in 1918, the viruses had continued to cause diseases in populations of pigs worldwide [9]. Much like human influenza [10], SIVs have been shown to be evolving by both antigenic drift and frequent reassortment [11,12]. Although infection of humans with swine influenza is a relatively uncommon occurrence, H1N1 and H3N2 of swine origin have been reported previously in North America, Europe and Asia, and have caused sporadic human infections over the past decades [13-18].

As one of the mainly representative subtypes, swine H1N1 influenza viruses have been reported widely throughout the world. The world-wide H1N1 pandemic in1918 also affected swine, and another classical swine H1N1 virus strain caused the well-known outbreak at Ft. Dix in New Jersey in 1976 [19]. In March and early April 2009, a new swine-origin influenza A (H1N1) Virus (S-OIV) emerged in Mexico and the United States, had developed into the first pandemic of the 21st century [20,21]. China is generally regarded as an epicenter of pandemic influenza viruses throughout history. In 2009 pandemic, 71 of the first 426 imported cases with confirmed cases of infection were from Guangdong province which was one of the most severely disaster area in China. It is documented that SIVs have become more human-like as well as more avian-like, the probability of generating human-adaptive viruses increase. Co-infections have allowed intermixing of these genomes to produce triple reassortants with genes derived from human, swine and avian influenza strains [22].

For a better understanding the influenza A virus lineages and evolution influenced by the 2009 H1N1, the study were focused on H1N1 SIVs in Guangdong province from 2008 to 2009. The whole genome sequence of each strain were analyzed, intending to learn more epidemiology information about the prevalence of SIVs, the possible correlations with the 2009 H1N1 strains in southern China, and provide useful guides for SI control.

## Results

### Virus isolation and identification

According to the detection results, eight isolates were positive for HA tests, subtyped to be H1N1 by RT-PCR assays. The eight H1N1 isolates obtained in this study were listed in Table 1.

**Table 1:** SIV strains isolated from Guangdong province of southern China.

Strain Name	PB1	PB2	PA	NA	HA	NP	NS	M
A/swine/Guangdong/11/2009/H1N1	HM145744^a^	HM145745	HM145743	HM145741	HM135403	HM145742	HM145747	HM145746
A/swine/Guangdong/09/2009/H1N1	HM210858	HM210859	HM210857	HM210854	HM210852	HM210855	HM210856	HM210853
A/swine/Guangdong/07/2009/H1N1	HM210866	HM210867	HM210865	HM210862	HM210860	HM210863	HM210864	HM210861
A/swine/Guangdong/06/2009/H1N1	HM215157	HM215158	HM215156	HM215153	HM215151	HM215154	HM215155	HM215152
A/swine/Guangdong/05/2009/H1N1	HM215165	HM215166	HM215164	HM215161	HM215159	HM215162	HM215163	HM215160
A/swine/Guangdong/09/2008/H1N1	HM215173	HM215174	HM215172	HM215169	HM215167	HM215170	HM215171	HM215168
A/swine/Guangdong/07/2008/H1N1	HM223592	HM223593	HM223591	HM223588	HM223586	HM223589	HM223590	HM223587
A/swine/Guangdong/02/2008/H1N1	HM223600	HM223601	HM223599	HM223596	HM223594	HM223597	HM223598	HM223595

^a ^accession number of each segmented gene

### Sequence identity analysis

The nucleotide sequence identities among the eight genes of each isolate were 99-100%, suggesting that the eight isolates might evolve from the same progenitor viruses. The genotypes and genetic origins of isolates were initially inferred from BLAST analysis and pairwise comparisons of each gene segment to the corresponding sequences of reference viruses. Taking into account the high levels of sequence identity between the eight isolates, isolate A/swine/Guangdong/11/2009 was used as the representative strain for blast search in the Genbank. Each of the eight genes of the eight isolates showed the highest nucleotide sequence identities to those of classical swine H1N1 virus, with homologies ranging from 98.8 to 99.6%.

### Phylogenetic relationship and distribution

**Figure 1:**
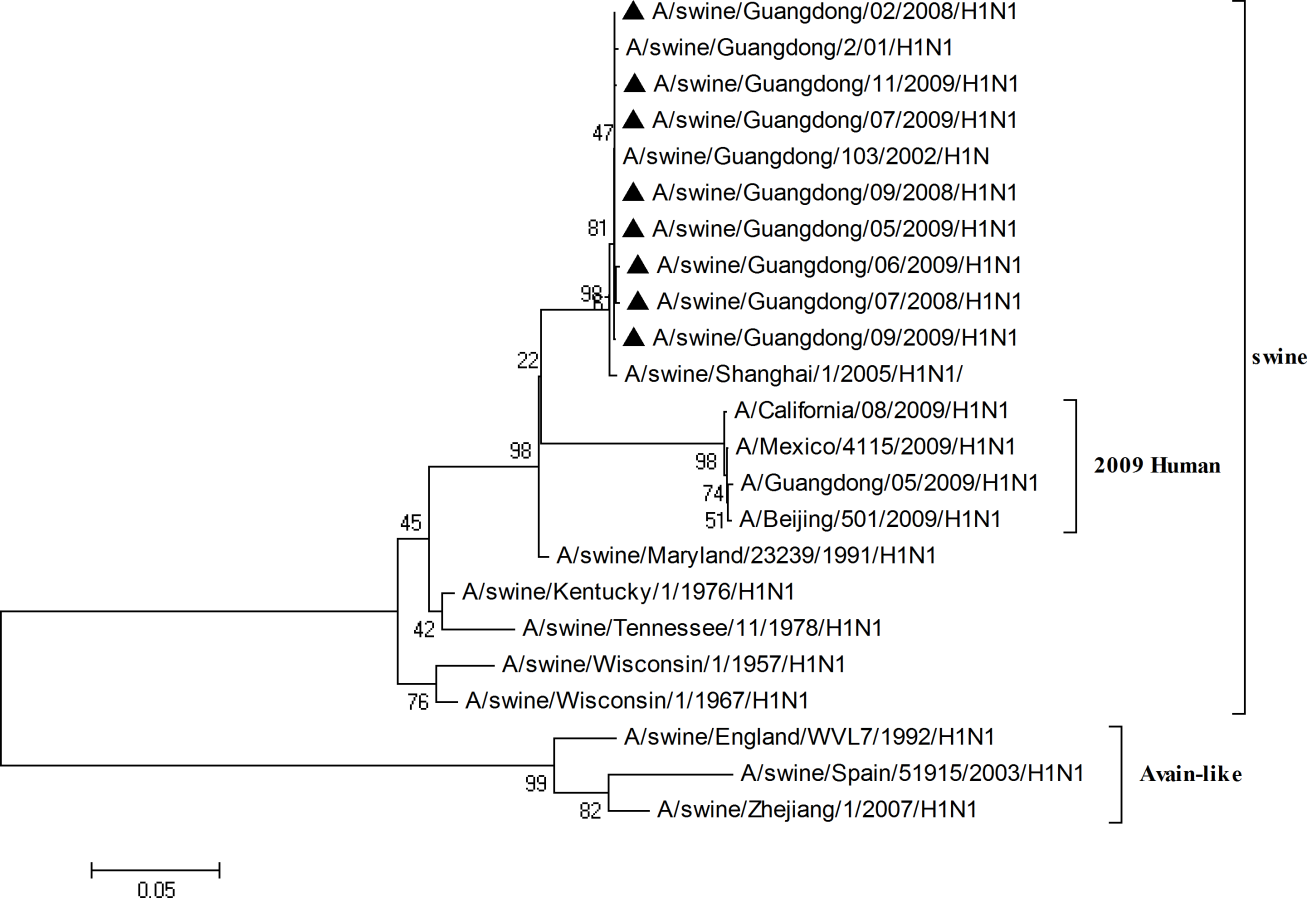
**Phylogenetic trees of the HA genes of influenza A viruses**. Analysis was based on the nucleotide sequences in open reading frames of the HA genes. The subtypes of the viruses were marked in the brackets. The virus marked with black triangle is the swine H1N1 virus isolated and sequenced in this study.

**Figure 2:**
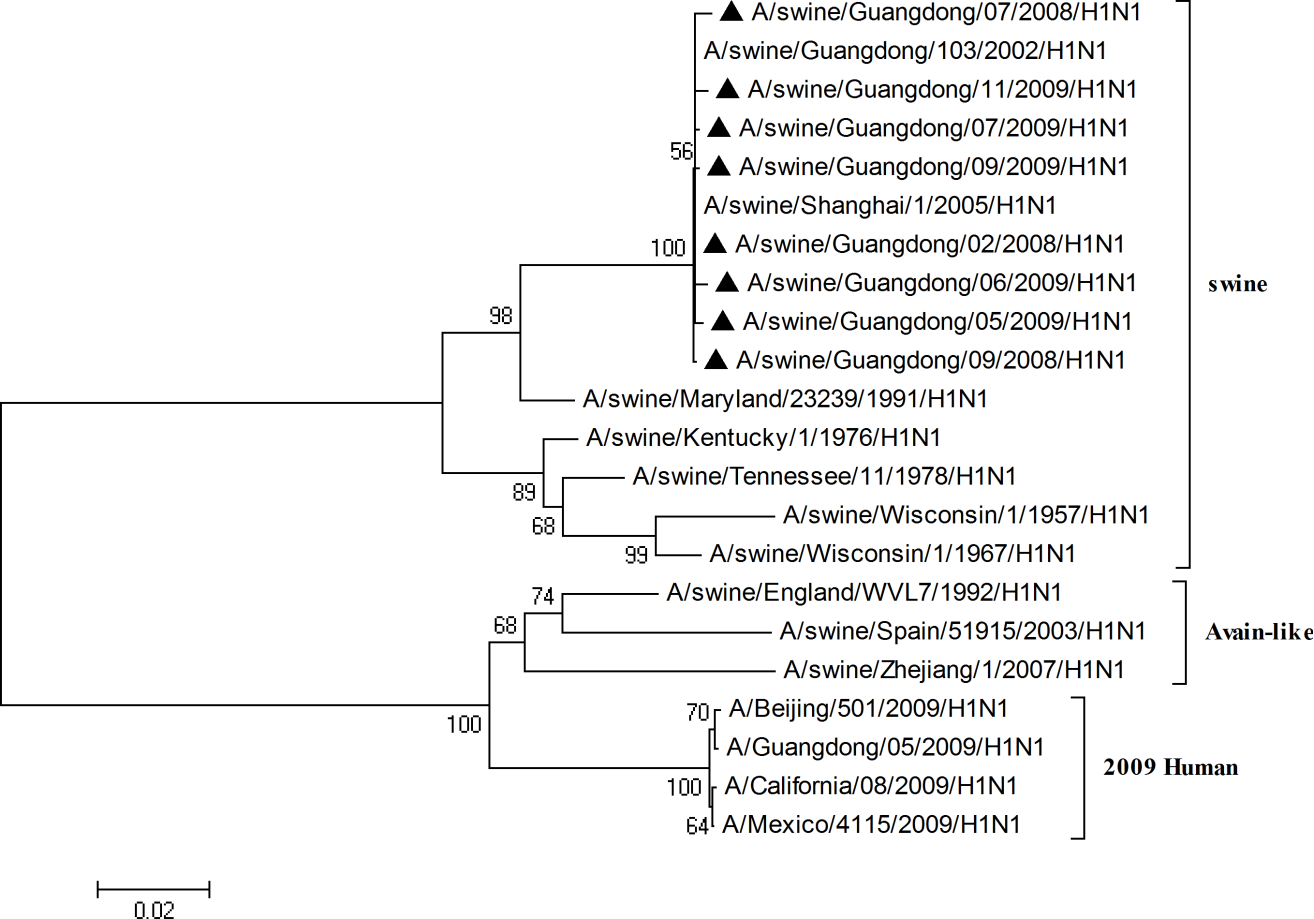
**Phylogenetic trees of the NA genes of influenza A viruses**. The virus marked with black triangle is the swine H1N1 virus isolated and sequenced in this study.

**Figure 3:**
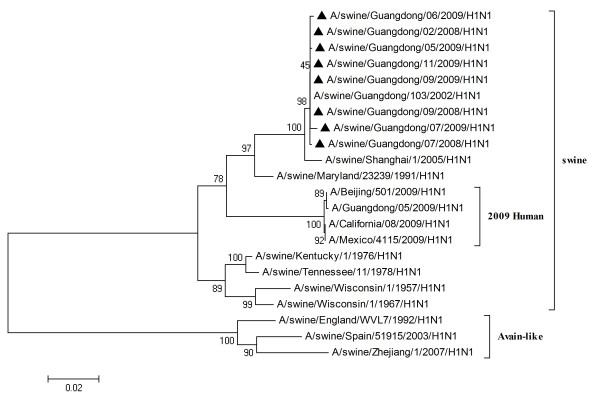
**Phylogenetic trees of the NP genes of influenza A viruses**. The virus marked with black triangle is the swine H1N1 virus isolated and sequenced in this study.

**Figure 4:**
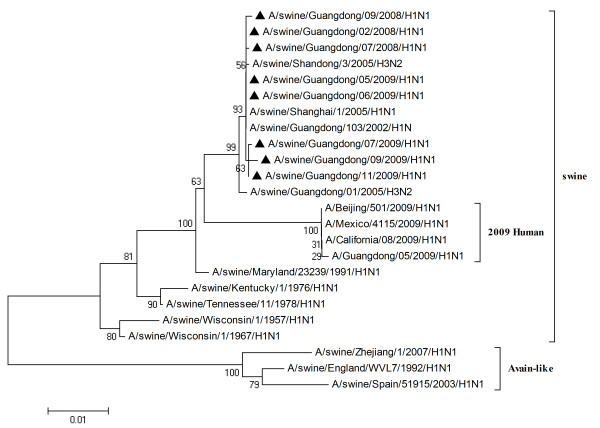
**Phylogenetic trees of the NS genes of influenza A viruses**. The virus marked with black triangle is the swine H1N1 virus isolated and sequenced in this study.

**Figure 5:**
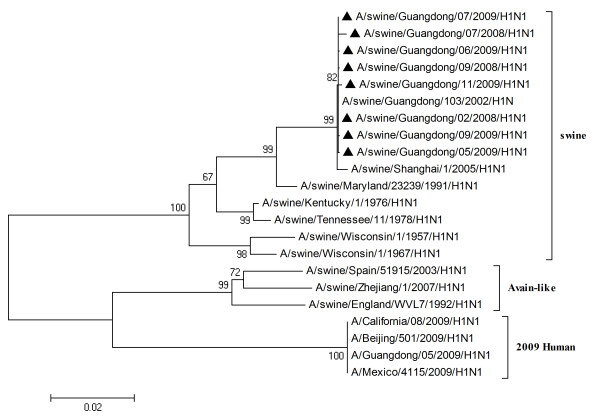
**Phylogenetic trees of the PB1 genes of influenza A viruses**. The virus marked with black triangle is the swine H1N1 virus isolated and sequenced in this study.

**Figure 6:**
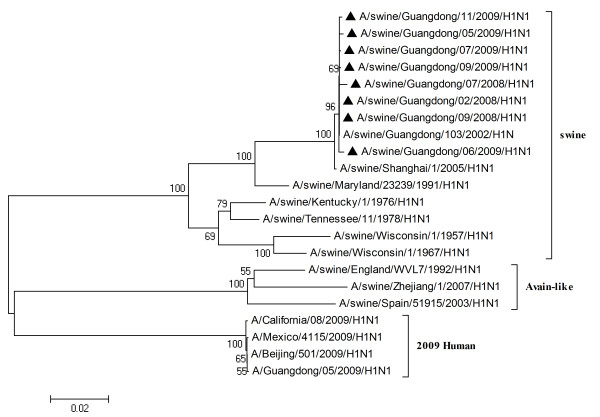
**Phylogenetic trees of the PB2 genes of influenza A viruses**. The virus marked with black triangle is the swine H1N1 virus isolated and sequenced in this study.

**Figure 7:**
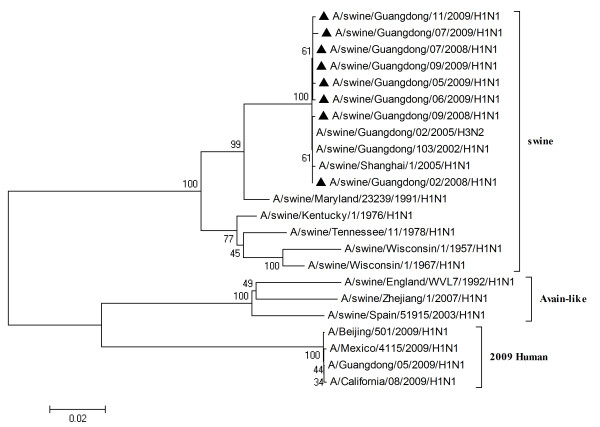
**Phylogenetic trees of the PA genes of influenza A viruses**. The virus marked with black triangle is the swine H1N1 virus isolated and sequenced in this study.

**Figure 8:**
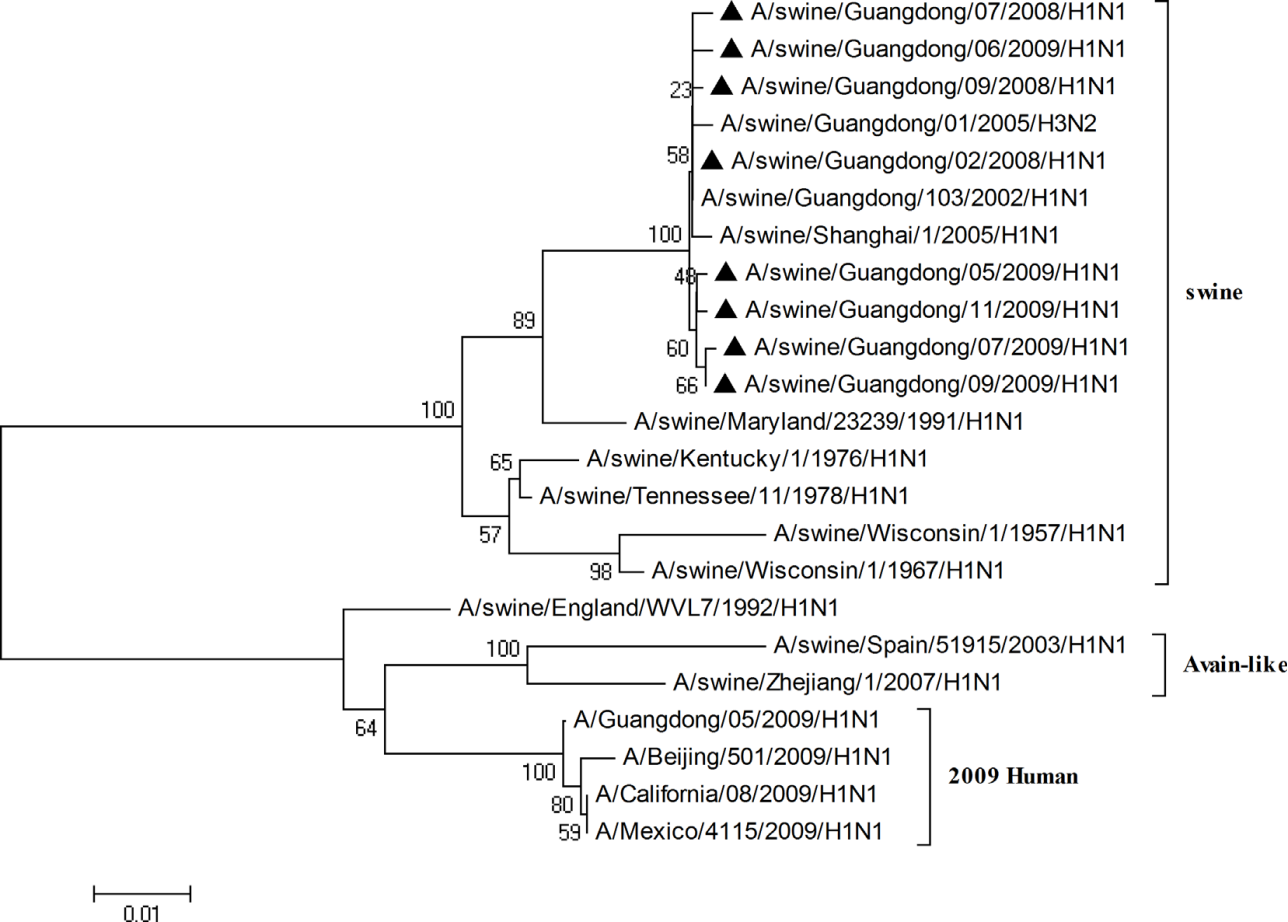
**Phylogenetic trees M genes of influenza A viruses**. The virus marked with black triangle is the swine H1N1 virus isolated and sequenced in this study.

To characterize the gene segments of the isolated viruses more precisely, the phylogenetic trees using the nucleotide sequences of eight genes were constructed with genes available in GenBank respectively. Three lineages correlative with 2009 human strains, classical swine strains and European swine strains were observed in the phylogenetic trees (Figures 1, 2, 3, 4, 5, 6, 7 &8). All the eight genes of the eight isolates, belonged to the same sublineage closely related to the classical swine H1N1 viruses, especially those isolated in China. The phylogenetic trees revealed that the HA, NP and NS segments of the isolated strains with the 2009 H1N1 strains were listed in the sister lineages of classical SIV (Figures 1, 3, 4), while the PB1, PB2, PA, NA and M genes of the eight genes were closed to the European H1N1 SIV (Figures 2, 5, 6, 7, 8). Meanwhile, two H3N2 viruses were also closely relative to the classical swine lineage of PA, M and NS, suggested the reassortment with the classical swine H1N1 viruses (Figures 4, 7, 8).

### Key site analyses

**Figure 9:**
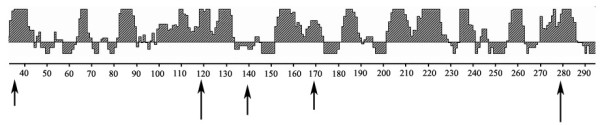
**Antigenic epitopes prediction (as showed by the upper peaks) of the HA1 of A/swine/Guangdong/11/2009 (represented the isolated strains)**. The mutated sites were pointed with the arrow.

The deduced amino acid sequences of the segemented genes were aligned and compared with the reference H1N1 strains. These isolates contained an amino acid motif PSIQSR↓G at the HA cleavage sites, which met the characteristic of low pathogenic influenza viruses. The predicted antigenic epitopes of the HA protein of the isolates were similar to the classical H1N1 SIVs, revealed five major changes located at the position 36, 120, 138, 170 and 278 between different lineages (Figure 9). The specific mutation was located at the position 36 between the isolated strains and the 2009 H1N1 strains, and the mutation located 138 compared to the avian like strains. In the study, all of the isolates displayed the mutations located at 120 and 170 between all of the avian like, 2009 H1N1-like and the classical H1N1 strains, but shared the same antigenic epitope at 278 with 2009 H1N1-like strains only.

The eight isolates maintained amino acids typical of swine viruses at residues 68E, 134A, 183P, 187D, 191L and 222G (according to H1 number) of HA genes, which were previously defined receptor-binding sites [23,24]. Only the European swine strains possessed D at position 68, A/swine/Zhejiang/1/2007/H1N1 possessed V at position 134, all the strains in this study possessed P at position 183, the 2009 H1N1 strains possessed S at position 183. The eight strains shared the same amino acid at the receptor-binding sites, which suggests that these isolates might have the same host range. As amino acid position 222 is in the receptor binding cavity, changes can potentially influence receptor binding of the influenza virus as well. In this study, the eight isolated strains, classical strains and a European strain possessed G at the position 222. The 2009 H1N1 strains possessed D, while the other European swine strains possessed E at the position 222.

The N-linked glycosylation sites are relatively conserved, and the number of potential glycosylation site (PGS) changes may influence the protein folding, oligomerization, quality control, sorting and transport [25]. Six potential glycosylation sites (PGSs) with an N-X-T/S motif (where X can be any amino acid except proline) were conserved at positions 10, 11, 23, 87, 276, and 287 in the HA1 and two were conserved at positions 154, 213 in the HA2 protein of A/Swine/Guangdong/11/2009. The isolated strains, 2009 H1N1 strains and the strains from Shanghai, Maryland and Kentucky of Classical swine had the same PGSs. The Wisconsin and Tennessee of Classical swine strains as the avian-like strains did not have the potential glycosylation sites at the 276 position.

A balance between the activities of HA in virus attachment and NA in virus release needs to be maintained for optimal viral replication [1]. No amino acid substitutions (H274Y and N294S) conferring resistance to oseltamivir were observed at the conserved residues in the NA proteins of the eight isolates, which suggest that they are sensitive to NA inhibitors. Eight PGSs in NA protein were found in the isolated strains and 2009 H1N1 strains, located at positions 44,58,63,68, 88,146,235 and 386 in linker region and the NA domain. The eight isolates shared the same PGSs distributions with the 2009 H1N1 strains, had two extra PGSs at the position 386 compared to the Wisconsin and Tennessee of Classical swine strains. The avian like H1N1 (A/swine/England/WVL7/1992/H1N1) carried different eight PGSs located amino acid positions 36, 44, 49, 54, 74, 132, 221 and 372 in the NA molecule.

Pathogenicity of influenza virus was polygenic. Internal proteins (M, PB1, PB2 and NS) also harbor determinants for host range and virulence, as demonstrated by genetic studies on avian-human reassortant viruses [26]. Anti-influenza drugs amantadine and rimantadine target the M2 protein, and the substitution S31N have been reported to confer resistance to these drugs in recent European swine viruses, including H1N1, H1N2, and H3N2 viruses [27]. Interestingly, the strains of same lineage had difference at the position of 31. The substitution S31N observed in the M2 protein genes of our isolates suggested not sensitive to the primary class of antiviral drugs.

PB1-F2 has been shown to localize to the mitochondria and induce apoptosis, enhance viral virulence in a mouse model [28,29]. Similar to most classical swine H1N1 viruses, the eight isolates contained truncated PB1-F2 proteins because of in-frame stop codon after residue 11. The highly conserved regions in the PB1 protein that may be functionally important and the amino acid residues at 375 might be critical for host specificity of the protein. Some difference appeared at the position of 375 between different strains. All of the eight isolates, 2009 H1N1 strains and Shanghai, Maryland strains possessed S at position 375, European swine strains were D or N, but other classical strains were G.

Previous study showed that residue 627 of the polymerase basic protein 2 (PB2) was associated with host range adaptation and recognized as one of the most important determinants [30-33]. In this study, each of the eight isolates possessed a K at position 627 of PB2 which was characteristic of mammalian influenza viruses, the classical swine strains possessed a K at position 627 of PB2, and the 2009 Human strains and avian-like strains were E.

The NS1 protein is also important in determining the pathogenicity of influenza A virus in different hosts. No deletions were observed at positions 80-84 of NS1 proteins in the isolates. All eight isolates possessed D rather than E at position 92 of NS1, which possesed a mutation might increase the virulence of H5N1 viruses in pigs. The exceptional A/swine/England/WVL7/1992/H1N1 (avian like H1N1) possessed T at position 92 of NS1.

## Discussion

Influenza virus infection is an important cause of respiratory disease among pigs throughout the swine producing regions of the world [34]. Pigs have been considered as mixing vessel for human and avian influenza viruses, allowing swine viruses to acquire avian and human virus gene segments to generate novel SIVs. In China, various subtypes of SIVs were present in pig populations. Four subtypes (H1N1, H1N2, H3N1, and H3N2) were circulating in pig populations, and the classical swine H1N1 viruses were the predominant influenza virus infecting and circulating in swine population [35]. In southern China, reassortment of classical swine H1N1 and human-like H3N2 viruses had been reported from 1976 to 1982 [36-38] and avian-like swine H1N1 viruses co-circulated with classical swine H1N1 viruses were also detected in pigs [39]. In last several years, many studies reported the co-circulation and reassortment of classical swine H1N1, avian-like H1N1 viruses and human-like H3N2 viruses in most area of China [37,38]. Some serological surveillance had also indicated that avian H4, H5 and H9 influenza viruses had been transmitted to pig populations in southeastern China [40,41].

Influenza virus genomes are well known to undergo antigenic drift or antigenic shift that enable escape from preexisting immunity and cause new outbreaks of influenza in animals and even humans [42-44]. In China, pigs have a short lifespan (approximately 6 months) and are not vaccinated with any type of swine influenza vaccine. Therefore, the viruses circulating in swine population might appear to have been under less immune selection pressure and all genes evolved more slowly than in humans and poultry [45]. The classical H1N1 remained highly conserved genetically until the 1990s, with only a limited number of nucleotide changes [46,47]. However, since the introduction of H3N2 viruses with genes of human and avian origin into pig populations and the resultant reassortment with classical H1N1 viruses, the dynamics of clinical disease and prevention of outbreaks has changed dramatically only recently.

In the present study, the phylogenetic and molecular property of the H1N1 SIV strains isolated at Guangdong province in southern China through the 2009 pandemic were described in detail. The whole genome sequences were analyzed, and the genetic relatedness of these isolates with 2009 H1N1 viruses, classical SIV and European SIV were studied. Based on the phylogenetic trees and homology of the nucleotide sequence of gene segments, the isolates were confirmed to be the classical swine virus. The nucleotide sequence identities among the eight genes were 99%-100%, indicated that the classical SIV H1N1 circulating in Guangdong of Southern China were resistant and might derive from the same ancestor. Interestingly, PA, M, and NS genes of two H3N2 strains from Guangdong province were located in similar lineages of the strains in the study, indicated the reassortment had occurred in classical H1N1 viruses. The isolated strains contained genes from the neighboring lineages with 2009 H1N1 which genetically distant from the avian-like clusters, provided further evidences that isolates in the study and the 2009 H1N1 viruses might be related with a finite extent.

Some key amino acids of the segmented gene have been implicated to play the vital role in host range adaptation, pathogenicity of influenza A virus in different hosts, receptor binding and antiviral drug resistance, and these traits are polygenic. Based on whole genome sequence analysis, the characteristics of each isolated strain were deeply and profoundly studied, revealed some resemblance and difference between different lineages. Generally, low pathogenic influenza viruses raise lowly concerning made these strains might have a greater opportunity to become widespread. The isolated strains had the same S at position 375 of PB1, as 2009 human strains, suggested that these strains should be raised more concern for the potentiality to infect human. It should be further studied to determine whether the PGSs changed in glucoprotein of the virion surface could alter the pathogenicity. Compared to the avian-like strains, the extra PGSs in HA protein at the 276 position supposed to enhance the pathogenicity for wide prevalence, and the additional PGSs in NA protein might reduce the ability of affinity between influenza virus and the host receptor as well as the release of the virus particles.

The special environment and lifestyle in southern China provide more chances for wild aquatic birds, domestic poultry, pigs and humans to contact closely, and create the opportunity for interspecies transmission and generation of new reassortment influenza viruses. Although the 2009 H1N1 has not been identified circulating endemically in swine, it is virtually impossible to prevent new outbreaks of influenza in human and animals. It had been recognized that influenza viruses in pigs may form the basis for newly emerging influenza pandemic after further adaptation to the human host or reassortment with other influenza A viruses. Therefore, carrying out swine influenza virus surveillance would be of great significance.

## Conclusions

In conclusion, the present study has demonstrated that the circulating SIV strains in commercial farms in southern China are mainly classical swine H1N1. In spite of the low pathogenicity, the strains in the study might have limit evolutionary correlations with the 2009 H1N1 viruses, but also provides the evidence that pigs served as reservoirs of ancestor influenza viruses for emergence of pandemic strains. We hope this study could provide some useful information for the prediction and preparedness of possibly future influenza pandemics.

## Materials and methods

### Viruses

Nasal swabs were collected from diseased swine in different pig farms from Guangdong province of southern China. Initial isolation was performed in10-day-old specific-pathogen-free embryonated chicken eggs. The Allantoic fluids were harvested after 3-4 days of incubation, and primarily diagnosed by HA test. The viruses were further confirmed and subtyped by 2 different multiplex RT-PCR as previously described. The remains were preserved at -70°C for the subsequent trials.

### RNA extraction and gene sequence

RNA was extracted from allantoic fluids using the QIAamp viral RNA Mini Kit (Qiagen Inc., Valencia, CA) according to the manufacturer's instructions. The full length of all eight genes (HA, NA, PA, PB1, PB2, NP, NS, M) of each isolates were amplified by RT-PCR using universal primer sets [48,49]. Reverse transcription polymerase chain reaction (RT-PCR) was carried out by PrimeScriptTM One-Step RT-PCR Kit (TaKaRa Biotechnology, Dalian, China) in 25 μl reaction volume containing 20 μl of RT-PCR PreMix (reaction buffer, dNTPs, 2 μl of enzyme mix), 2 μl of extracted viral RNA and the specific primer pair. The PCR products were cloned into pMD19-T vector (TaKaRa Biotechnology, Dalian, China) for later sequencing (AuGCT Biotechnology, Beijing, China).

### Sequence analysis and phylogenetic analysis

Sequences were assembled using the Lasergene Seqman package IV (DNAStar 6.0) and aligned using multiple sequence alignment tool CLUSTAL W 1.83. The phylogenetic trees were constructed using the MEGA 4.1 software with neighbor-joining method [50] and each tree was produced using a consensus of 1000 bootstrap replicates. All the other H1N1 viral sequences of eight genes for reference were from the GenBank database.

### Nucleotide sequence accession numbers

The segmented sequences determined in this study were available from GenBank database and the corresponding accession numbers were listed in Table 1.

### Antigenicity and surface probability analysis

DNA Star software was used to analyze the antigenicity epitopes of the HA proteins of each isolates, using Jameson-Wolf methods (DNAStar Inc., Madison, WI, USA). Mature peptide sequences of HA and NA genes of the H1N1 SIV strains were submitted for prediction of potential N-glycosylation sites using the CBS on-line server 1.0 (http://www.cbs.dtu.dk/services/NetNGlyc/).

## Competing interests

The authors declare that they have no competing interests.

## Authors' contributions

YL and JJ carried out most of the experiments and wrote the manuscript, and should be considered as first authors. QX critically revised the manuscript and the experiment design. JW, HS, CC, FC, CX, YC, JM and YB helped with the experiment. All of the authors read and approved the final version of the manuscript.
